# Exploring 3D elastic-wave scattering at interfaces using high-resolution phased-array system

**DOI:** 10.1038/s41598-022-12104-9

**Published:** 2022-05-25

**Authors:** Yoshikazu Ohara, Marcel C. Remillieux, Timothy James Ulrich, Serina Ozawa, Kosuke Tsunoda, Toshihiro Tsuji, Tsuyoshi Mihara

**Affiliations:** 1 Department of Materials Processing, Tohoku University, Sendai, Miyagi 980-8579 Japan; 2 Los Alamos National Laboratory, Los Alamos, NM 87545 USA

**Keywords:** Mechanical engineering, Imaging techniques, Characterization and analytical techniques, Acoustics, Ultrasound

## Abstract

The elastic-wave scattering at interfaces, such as cracks, is essential for nondestructive inspections, and hence, understanding the phenomenon is crucial. However, the elastic-wave scattering at cracks is very complex in three dimensions since microscopic asperities of crack faces can be multiple scattering sources. We propose a method for exploring 3D elastic-wave scattering based on our previously developed high-resolution 3D phased-array system, the piezoelectric and laser ultrasonic system (PLUS). We describe the principle of PLUS, which combines a piezoelectric transmitter and a 2D mechanical scan of a laser Doppler vibrometer, enabling us to resolve a crack into a collection of scattring sources. Subsequently, we show how the 3D elastic-wave scattering in the vicinity of each response can be extracted. Here, we experimentally applied PLUS to a fatigue-crack specimen. We found that diverse 3D elastic-wave scattering occurred in a manner depending on the responses within the fatigue crack. This is significant because access to such information will be useful for optimizing inspection conditions, designing ultrasonic measurement systems, and characterizing cracks. More importantly, the described methodology is very general and can be applied to not only metals but also other materials such as composites, concrete, and rocks, leading to progress in many fields.

## Introduction

Wave phenomena at interfaces between two media are essential in many research and industrial fields. Among them, the scattering of elastic and electromagnetic waves at discontinuous interfaces in an elastic medium, such as cracks, is applicable to nondestructive inspection for the safety and reliability of structures and mechanical components. Elastic waves are strongly scattered at cracks, and hence, the use of elastic-wave scattering has been extensively studied for crack inspection^1,2^. Specifically, recent progress in ultrasonic phased-array (PA) imaging^3,4^ is noteworthy. PA imaging typically uses a linear array transducer^5,6^ composed of multiple rectangular piezoelectric elements, providing 2D ultrasonic images. Since one can intuitively recognize defects from PA images, it has been widely used in industrial fields. Moreover, the signal-to-noise ratio (SNR) has also been improved through the development of various types of PAs in research fields^7–19^. Given that ultrasonic crack inspections utilize the scattering phenomena at cracks, understanding the elastic-wave scattering at cracks is indispensable. Thus far, most studies on ultrasonic scattering at cracks have been limited to two dimensions^18,20–23^. On the other hand, an actual crack is composed of multiple scattering sources since crack faces have microscopic asperities^24^ and parts of the faces are sometimes in contact^25^. Hence, the ultrasonic scattering at cracks is very complex in three dimensions because of the superposition of ultrasonic scattering at individual scattering sources within a crack, which can exhibit different scattering behaviors. To elucidate the ultrasonic scattering phenomena at such interfaces, a method for investigating 3D ultrasonic scattering behaviors at each scattering source is indispensable. Although some studies on 3D ultrasonic scattering have been reported^26,27^, the bottleneck in challenging the complexity of 3D ultrasonic scattering at cracks was the lack of a high-resolution 3D ultrasonic imaging method for three-dimensionally resolving multiple scatterers within a single crack.

To achieve 3D ultrasonic imaging, a PA system using a matrix array transducer^3^ has long been a promising approach. A matrix array transducer is typically composed of small square piezoelectric elements. Given that the number of elements in linear array transducers ranges from 32 to 128, the number of elements in a matrix array transducer should be at least 1024 (i.e., 32 × 32) to realize sufficient image resolution. However, such PA systems have prohibitive costs and technical difficulties. Hence, the number of piezoelectric 2D array transducers is typically less than 256 for nondestructive testing (NDT) applications^28–31^, although state-of-the-art medical ultrasonic imaging has used a piezoelectric matrix array transducer with ultra-multiple elements, e.g., 1024 elements^32,33^.

On the other hand, we previously proposed a method of combining a single-element piezoelectric transducer and a linear array transducer to image closed cracks^7,8,16,34,35^. Here, a piezoelectric transmitter was used to provide the large-amplitude incidence required for nonlinear ultrasonics^36^. A linear array transducer was employed as a receiver for imaging. As an alternative, we also proposed combining a single-element piezoelectric transducer and the 1D scanning of a laser Doppler vibrometer (LDV)^37^. Note that LDV scanning can simulate the reception at each element of a linear array transducer. In contrast to the fixed number of elements of a piezoelectric linear array transducer, the number of receiving points can be increased as desired. The scan pitch corresponding to an element pitch for a piezoelectric array transducer can also be flexibly changed. Moreover, the reception bandwidth of an LDV is broad (e.g., DC to 20 MHz). This enables the arbitrary selection of frequency only by changing the transmitter and the simultaneous reception of the fundamental wave and nonlinear signals with various frequencies in nonlinear ultrasonics. However, the method has only been tested for 2D imaging^37,38^. To open up a new avenue to 3D PA imaging, we previously proposed a PA system, the piezoelectric and laser ultrasonic system (PLUS)^39^. PLUS combines a piezoelectric transmitter and a 2D scan of an LDV to simulate a 2D matrix array with ultra-multiple elements. We demonstrated the high-resolution 3D imaging capability for a flat bottom hole (FBH) and for stress corrosion cracking (SCC)^39^. However, the analysis of the 3D ultrasonic scattering at cracks has yet to be realized, although understanding such phenomena is essential for optimizing inspection conditions, designing ultrasonic measurement systems, and characterizing cracks.

In this study, we propose a method for exploring the 3D elastic-wave scattering at interfaces based on the PLUS. We first describe the principle of the 3D phased-array imaging method using PLUS, for three-dimensionally resolving scatterers within a single crack, followed by the formulation. We propose a method based on PLUS for extracting the 3D elastic-wave scattering in the vicinity of each response from a dataset of waveforms used for 3D imaging. We experimentally show the effectiveness of the proposed methodology for a fatigue-crack specimen.

This paper is organized as follows. In the “Methods” section, the principles of PLUS and the proposed 3D elastic-wave scattering analysis method are introduced and illustrated on the experimental example of a simple defect, i.e., an FBH. Then, the performance of the method is evaluated on experimental data obtained in a fatigue-crack specimen in the “Results” section. The generality and extensive future possibilities of the 3D elastic-wave scattering analysis method are considered in the “Discussion” section.

## Methods

### High-resolution 3D imaging method using PLUS

**Figure 1:**
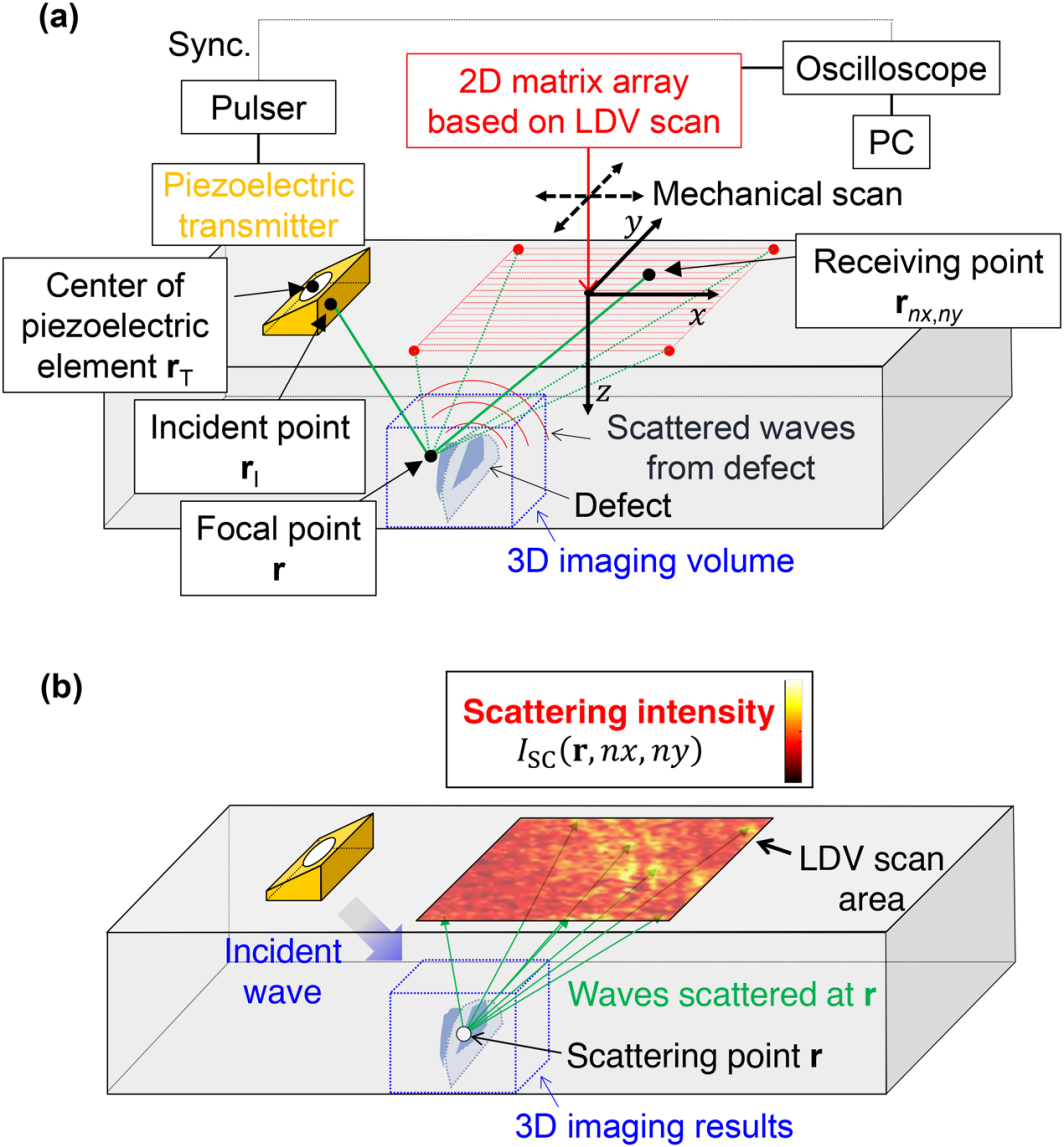
(Color online) Schematic illustrations of PLUS and 3D ultrasonic scattering analysis method. (**a**) 3D ultrasonic phased-array imaging system, PLUS. (**b**) Method for examining the 3D ultrasonic scattering intensity ${I}_{\mathrm{SC}}\left(\mathbf{r}, nx, ny\right)$ for the response in the vicinity of **r** in the 3D imaging results. The distribution of the scattering intensities over the scan area is directly extracted from the received waves by pinpointing the temporal region of the waves scattered in the vicinity of $\mathbf{r}$ by the calculation of the arrival time at ${\mathbf{r}}_{nx,ny}$.

To propose a 3D elastic-wave scattering analysis method based on PLUS, we first describe the principle of PLUS for high-resolution 3D imaging. PLUS comprises a single-element piezoelectric transmitter and a 2D matrix array receiver based on the 2D scanning of an LDV. As illustrated in Fig. 1a, a piezoelectric transducer mounted on a wedge emits an ultrasonic wave into a specimen. The wave mode and incident angle for the oblique incidence can be selected using a suitable wedge. When defects exist within a region irradiated by the ultrasonic wave, they can be ultrasonic scattering sources. The scattered waves are then received at a point on the top surface by an LDV, which can measure an out-of-plane displacement in a small laser-irradiation area. The received wave is transferred through an oscilloscope to a PC. The process is repeated while moving the receiving point over a scan area. Here, the LDV receiving points correspond to the elements of a piezoelectric array transducer. Hence, a 2D matrix array with ultra-multiple elements can be readily realized by increasing the number of scanning points, e.g., to the order of thousands, which is impossible for piezoelectric array transducers. In this way, a complete dataset of the received waves is stored on a PC. Subsequently, the dataset is post-processed to create 3D images within a volume of interest (VOI) following an imaging algorithm described later. Note that an LDV has a broad reception bandwidth, enabling the use of an arbitrary frequency range in the same system simply by changing the piezoelectric transmitter. Furthermore, in contrast to laser ultrasonics^40–42^ using lasers for both emission and reception, a large-amplitude ultrasonic incidence with a piezoelectric transmitter can compensate for the low SNR of the LDV due to the small laser irradation spot. This enables high-SNR imaging, which is essential for research and industrial applications.

We next describe the imaging algorithm for PLUS. The center of the LDV scan area is defined as the origin ***S***_0_ in *x*–*y*–*z* Cartesian coordinates. The propagation time in the wedge of the transmitter is calculated as

1 $${t}_{\mathrm{W}}=\frac{\left|{\mathbf{r}}_{\mathrm{T}}-{\mathbf{r}}_{\mathrm{I}}\right|}{{V}_{\mathrm{W}}} ,$$

where **r**_T_ is the center of the piezoelectric disk, **r**_I_ is the incident point at the specimen's top surface, and *V*_W_ is the longitudinal wave speed in the wedge. Here we fixed **r**_I_ assuming the plane-wave propagation in the wedge because of the small propagation distance in the wedge. The propagation time from the transmitter through a point **r** to ***S***_0_ is given by

2 $${t}_{0}\left(\mathbf{r}\right)={t}_{\mathrm{W}}+\frac{\left|{\mathbf{r}}_{I}-\mathbf{r}\right|}{{V}_{i}}+\frac{\left|\mathbf{r}\right|}{{V}_{j}} ,$$

where ${V}_{i}$ and ${V}_{j}$ are the speeds of the incident and scattered waves, respectively, and *i* and *j* are either L for a longitudinal wave or T for a transverse wave. Here we assume a spherical-wave incidence from **r**_I_ in the sample. Note that one can select the approximation of the plane-wave incidence^43–45^ when the propagation distance in a sample is shorter than the near-field length^1,2^. The difference in the approximations has been reported in the Supplementary Material. Likewise, the propagation time from the transmitter through **r** to the receiving point **r**_*nx*,*ny*_ is calculated as

3 $${t}_{nx, ny}\left(\mathbf{r}\right)={t}_{\mathrm{W}}+\frac{\left|\mathbf{r}-{\mathbf{r}}_{I}\right|}{{V}_{i}}+\frac{\left|{\mathbf{r}}_{nx,ny}-\mathbf{r}\right|}{{V}_{j}} ,$$

where *nx* and *ny* are the indices of the receiving point in the *x*- and *y*-directions, respectively. The delay law for each receiving point is obtained by subtracting ${t}_{0}\left(\mathbf{r}\right)$ from ${t}_{nx, ny}\left(\mathbf{r}\right)$;

4 $${\Delta t}_{nx, ny}\left(\mathbf{r}\right)={t}_{nx, ny}\left(\mathbf{r}\right)-{t}_{0}\left(\mathbf{r}\right)=\frac{\left|{\mathbf{r}}_{nx,ny}-\mathbf{r}\right|-\left|\mathbf{r}\right|}{{V}_{j}} .$$

As the imaging algorithm, delay-and-sum processing is used here. Assuming that the wave ${u}_{nx,ny}(t)$ is received at ${\mathbf{r}}_{nx,ny}$, the waveform after delay-and-sum processing for **r** is calculated as

5 $$U\left(\mathbf{r}, t\right)=\frac{1}{{N}_{x}{N}_{y}}\sum\limits_{nx=1}^{{N}_{x}}\sum\limits_{ny=1}^{{N}_{y}}{u}_{nx,ny}\left(t-\Delta {t}_{nx,ny}\left(\mathbf{r}\right)\right) ,$$

where ${N}_{x}$ and ${N}_{y}$ are the numbers of receiving points in the *x*- and *y*-directions, respectively. The scattering intensity for a voxel within a VOI is calculated as the root mean square (RMS) of $U\left(\mathbf{r}, t\right)$,

6 $$I\left(\mathbf{r}\right)={\left(\frac{1}{\Delta \tau }{\int }_{{t}_{0}(\mathbf{r})}^{{t}_{0}(\mathbf{r})+\Delta \tau }{U}^{2}\left(\mathbf{r}, t\right) dt\right)}^{1/2} ,$$

where $\Delta \tau$ is the burst length. Thus, a volumetric 3D image is obtained by repeating the above postprocessing for all **r** within a VOI. For visibility, the 3D images can be displayed only when $I\left(\mathbf{r}\right)$ is above a threshold, while those less than the threshold were treated as transparent. Note that a B-scan image in an arbitrary plane within the VOI can be extracted from $I\left(\mathbf{r}\right)$.

### 3D elastic-wave scattering analysis method based on PLUS

We propose a method based on PLUS for exploring 3D ultrasonic scattering at interfaces such as cracks. As illustrated in Fig. 1b, the aim is to examine the scattering intensity for the response at **r** over the scan area directly from the raw received waves to explore the 3D scattering phenomena. In the delay-and-sum algorithm for the 3D imaging, the received waves are delayed and summed following Eqs. (1)–(6). Here, to examine the 3D scattering behavior, the response in the vicinity of **r** for the analysis is selected in the 3D imaging results obtained by PLUS. Then, the arrival time ${t}_{nx, ny}\left(\mathbf{r}\right)$ at ${\mathbf{r}}_{nx,ny}$ of the waves scattered in the vicinity of **r** is calculated from Eq. (3) over the LDV scan area. Finally, the 3D scattering behavior for the response in the vicinity of **r** can be examined as the scattering-intensity distribution

7 $${I}_{\mathrm{SC}}\left(\mathbf{r}, nx, ny\right)={\left(\frac{1}{\Delta \tau }{\int }_{{t}_{nx, ny}\left(\mathbf{r}\right)}^{{t}_{nx, ny}\left(\mathbf{r}\right)+\Delta \tau }{{u}_{nx,ny}}^{2}\left(t\right) dt\right)}^{1/2}$$

over the scan area, where $\Delta \tau$ is the burst length. Although one needs to care about the overlap of scattered waves for multiple scatterers in the time window as described in detail later, the methodology proposed here can explore the main feature of 3D scattering behaviors for each response of the interfaces.

### Fundamental verification in an FBH specimen

**Figure 2:**
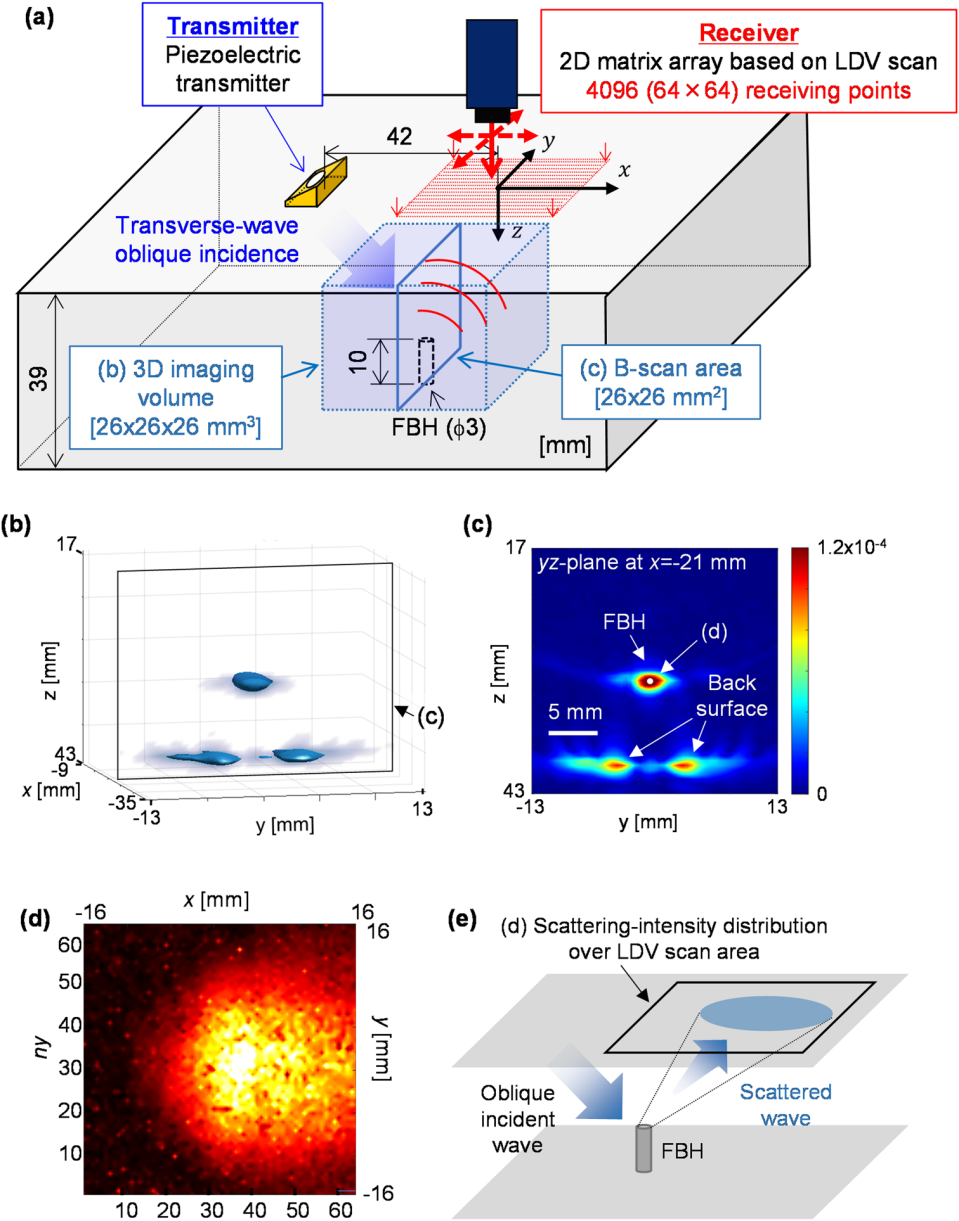
(Color online) Experimental configurations with PLUS for 3D imaging of the FBH and the imaging results. (**a**) Experimental configuration with PLUS with 4096 receiving points for imaging the FBH. (**b**) 3D images. (**c**) B-scan (*yz*-plane at *x* =  − 21 mm) image extracted from (**b**). (**d**) Scattering intensity ${I}_{\mathrm{SC}}\left(\mathbf{r}, nx, ny\right)$ from the FBH response at the point indicated as a white circle in (**c**). (**e**) Schematic illustrating the waves propagating from the FBH top.

To validate the proposed methodology, we prepared an aluminum alloy (A5052) specimen^39^ with a simple defect, i.e., an FBH (Fig. 2a). The FBH had a diameter of 3 mm and a height of 10 mm, and the thickness of the specimen was 39 mm. As shown in Fig. 2a, a piezoelectric transmitter (5 MHz, ϕ12.8 mm) was coupled to a wedge designed to input transverse waves with an oblique incidence of 45°. The longitudinal wave speed *V*_W_ in the wedge was 2730 m/s, which was used in Eq. (1). The transverse wave speed *V*_T_ in the specimen was measured to be 3165 m/s, which was used as *V*_*i*_ and *V*_*j*_ in Eqs. (2)–(4). The transmitter was excited by a square wave at a voltage of − 200 V. An LDV (OFV505, Polytec) received scattered waves at a point on the specimen's top surface. Note that the LDV used has a flat reception bandwidth ranging from DC to 20 MHz. The received signal digitized at a sampling rate of 250 MS/s was averaged 64 times with an oscilloscope and then transferred to a PC. We repeated the data acquisition process while scanning the LDV over the scan area composed of 4096 receiving points (i.e., ${N}_{x}={N}_{y}=64$)^39^, which cannot be realized using commercial piezoelectric 2D matrix array transducers. Here, the pitch between the adjacent receiving points was 0.5 mm in both the *x*- and *y*-directions. The VOI corresponding to the 3D imaging volume was 26 × 26 × 26 mm^3^. The pitch of the imaging grid composed of multiple $\mathbf{r}$ in Eq. (6) was set to 0.5 mm in the *x*-, *y*-, and *z*-directions within the VOI.

Figure 2b shows a bird’s-eye view of the 3D imaging results obtained by PLUS. In addition to the responses at the back surface, a strong response appeared at the correct position at the top of the FBH. The FBH response was in good agreement with the actual diameter and height. This shows that PLUS with ultra-multiple receiving points is useful for obtaining high-resolution 3D images^39^. To determine the point for the 3D scattering analysis, we extracted the B-scan (*yz*-plane at *x* =  − 21 mm) image (Fig. 2c) from the 3D image Fig. 2b). For the point shown as the white circle at the center of the FBH response in Fig. 2c, we examined the scattering intensity following Eq. (7). We found that the waves scattered at the FBH top were concentrated in the area surrounded by the white dotted circle, as shown in Fig. 2d. This result is reasonable because the shape of the FBH top is a circle. Also, the area with the strong responses in Fig. 2d can be explained by the reflection of the incident wave on the FBH top with the reflection angle equal to the incident angle, as illustrated in Fig. 2e. This implies that the scattering intensity obtained here was not only from the point at the center of the FBH top but also the area in the vicinity of the center. That is, the superposition of the scattered waves in the vicinity of the center of the FBH top can be obtained by the proposed method. The analysis of scattering-intensity distribution could give us the required scan area for a defect, which may lead to optimizing imaging conditions and designing a piezoelectric array transducer. Thus, we confirmed the fundamental validity of the algorithm for 3D ultrasonic scattering analysis.

## Results

### Demonstration of high-resolution 3D imaging capability of PLUS in a fatigue-crack specimen

**Figure 3:**
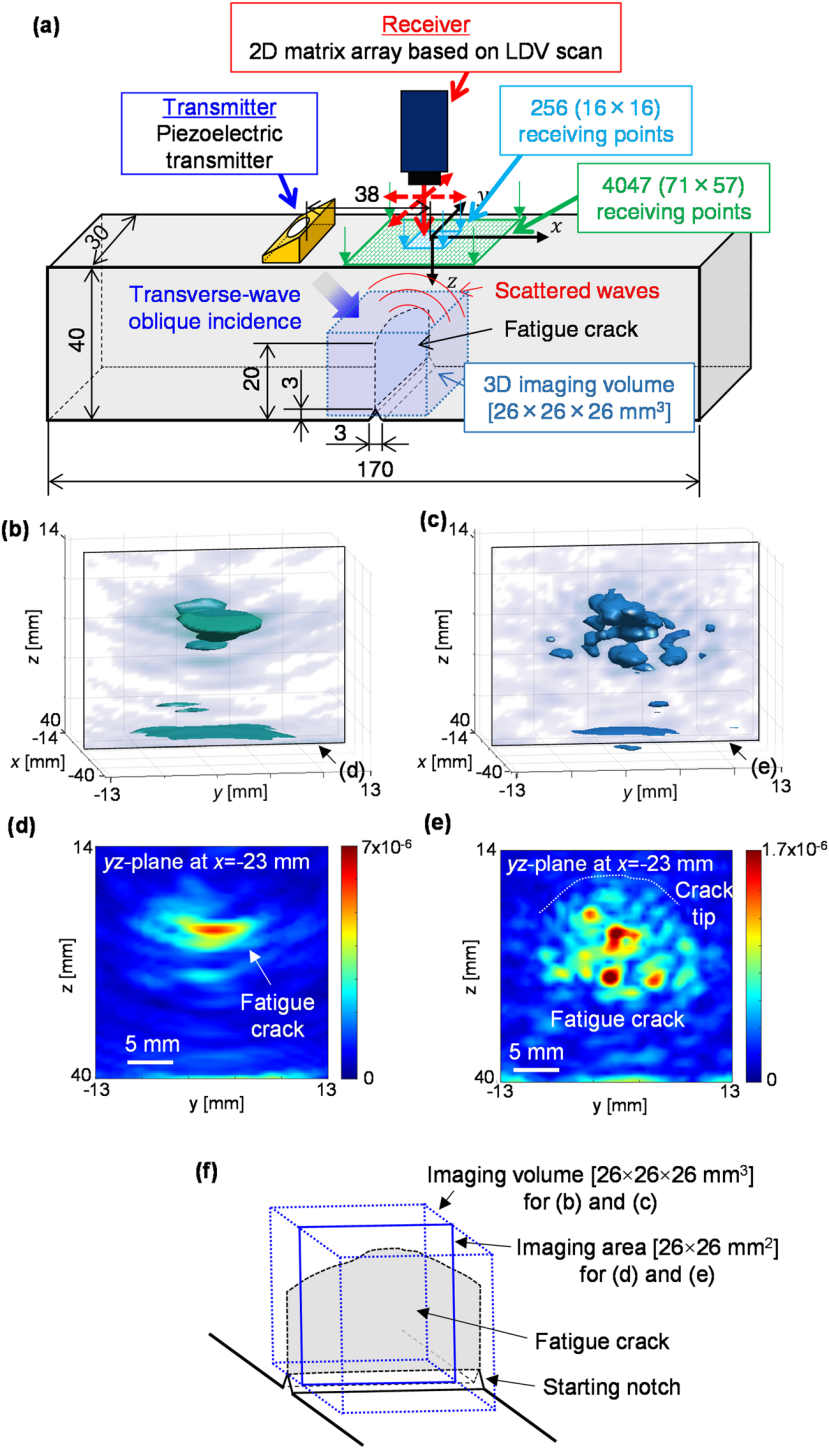
(Color online) Experimental configurations with PLUS with 256 and 4047 receiving points and the imaging results of a fatigue crack. (**a**) Experimental configurations with PLUS with 256 and 4047 receiving points for 3D imaging of the fatigue crack. (**b**, **c**) 3D images obtained with 256 and 4047 receiving points, respectively. (**d**, **e**) B-scan (*yz*-plane at *x* =  − 23 mm) images extracted from (**b**) and (**c**), respectively. (**f**) Schematic illustrating the relationship between the fatigue crack and the imaging region.

To demonstrate the efficacy of utilizing ultra-multiple elements of PLUS, we formed a fatigue crack in an aluminum alloy (A7075) specimen (Fig. 3a) by performing a three-point bending test. Under the fatigue conditions of a maximum stress intensity factor of 5.3 MPa m^1/2^ and a minimum stress intensity factor of 0.6 MPa m^1/2^^7^, we extended the crack from a starting notch to a depth of approximately 20 mm on the side surfaces. According to fracture mechanics^46^, the fatigue crack should have different depths (i.e., the crack size in the *z*-direction) inside the specimen. The crack depth should be largest around the center of the *y*-direction^47,48^. The transverse wave speed *V*_T_ in the specimen was measured to be 3080 m/s.

As shown in Fig. 3a, we used the same transmitter employed in the experiment for imaging the FBH (Fig. 2a). The transmitter was excited by a square wave at a voltage of − 150 V. The scattered waves were received at a point on the specimen's top surface by LDV and stored in a PC through the oscilloscope with the same sampling rate and the number of averaging as the previous experiment. Here, we selected two scanning conditions to examine the importance of utilizing ultra-multiple elements for the high-resolution 3D imaging of fatigue cracks. One condition employed 256 receiving points (i.e., 16 × 16) to simulate the maximum number of elements for a piezoelectric 2D matrix array^28–31^. The other condition employed 4047 receiving points (i.e., 71 × 57) to demonstrate the high-resolution 3D imaging capability of PLUS. Note that 4047 is an unachievable number of elements for piezoelectric array transducers. Here, the pitch between the adjacent receiving points was fixed to 0.5 mm in the *x*- and *y*-directions for both 256 and 4047 receiving points. The center of the scan area was identical for both conditions. The VOI was 26 × 26 × 26 mm^3^. The pitch of the imaging grid was set to 0.5 mm pitch in the *x*-, *y*-, and *z*-directions within the VOI.

Figure 3b, c show the 3D images obtained by PLUS with 256 (i.e., 16 × 16) and 4047 (i.e., 71 × 57) receiving points, respectively. Here, the 3D images showing the responses above a threshold were displayed with the semitransparent B-scan (*yz*-plane at *x* =  − 23 mm) images, which were extracted from the 3D images and correspond to the plane having the fatigue-crack faces. The B-scan images are also shown as opaque images in Fig. 3d, e. Note that B-scan (*xz*-plane) images^7,15,17,19,48,49^ obtained with a linear array transducer are typically perpendicular to the plane in which the crack exists. Such B-scan (*xz*-plane) images cannot resolve the crack in the *y*-direction because of the elevation aperture of a linear array transducer. In contrast, arbitrary planes within the VOI can be extracted from the 3D images since PLUS utilizes the 2D matrix array having ultra-multiple receiving points. Hence, we extracted the B-scan (*yz*-plane at *x* =  − 23 mm) images to examine the crack geometry in detail, as shown in Fig. 3d, e. Figure 3f illustrates the relationship between the fatigue crack and the imaging region for Fig. 3b–e.

For the matrix array with 256 receiving points, the fatigue crack appeared as a single response in Fig. 3b, d. However, the detail of the fatigue crack was not resolved because of the low resolution resulting from the limited number of receiving points. This suggests that 256 receiving points were insufficient to realize high-resolution 3D imaging of the fatigue crack.

For the matrix array with ultra-multiple elements, i.e., 4047 receiving points, the fatigue crack was visualized with high resolution as a collection of the multiple scattering points in Fig. 3c, e. Moreover, the outline of the fatigue-crack geometry was obtained by connecting the responses at the fatigue crack tips in the B-scan (*yz*-plane) image, as denoted by a white dotted curve in Fig. 3e. The geometry showing the maximum depth around the center in the *y*-direction was in good agreement with the fracture mechanics^46–48^. Note that a PA using a linear array transducer cannot obtain such images because of the low resolution in the *y*-direction due to the elevation aperture of the linear array transducer, as described above. The maximum crack depth in Fig. 3e was larger than that in Fig. 3d. This suggests that PLUS with ultra-multiple receiving points can improve the measurement accuracy of crack depths. Most importantly, the image resolution was much higher for 4047 receiving points than for 256 receiving points. For the further demonstration of the 3D imaging capability of PLUS for multiple scatterers, see the Supplementary Material. Thus, PLUS with ultra-multiple receiving points can resolve a fatigue crack into multiple scattering sources, which is indispensable for exploring 3D ultrasonic scattering phenomena at interfaces.

### Exploration of 3D ultrasonic scattering at the crack by PLUS

**Figure 4:**
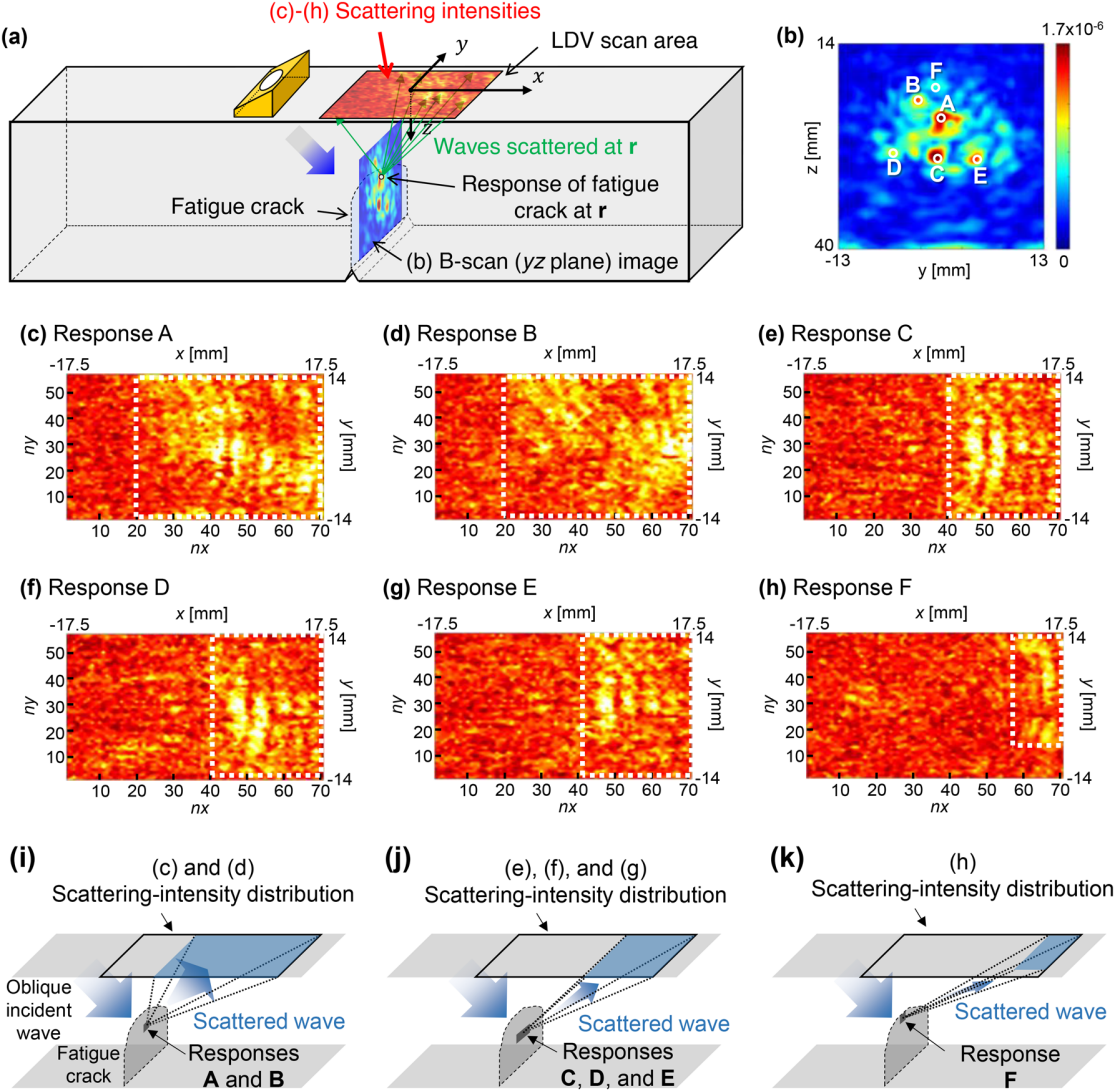
(Color online) Scattering-intensity distribution ${I}_{\mathrm{SC}}\left(\mathbf{r}, nx, ny\right)$ for various responses within the fatigue crack. (**a**) Schematic illustrating the geometric relationship between (**b**) and (**c**)–(**h**), (**b**) B-scan (*yz*-plane at *x* =  − 23 mm) images obtained with 4047 receiving points (Fig. 3e). (**c**)–(**h**) Scattering intensities ${I}_{\mathrm{SC}}\left(\mathbf{r}, nx, ny\right)$ from the crack responses at A (**r** = [*x* =  − 23 mm, *y* = 0.5 mm, *z* = 23 mm]), B (**r** = [*x* =  − 23 mm, *y* = 8 mm, *z* = 24 mm]), C (**r** = [*x* =  − 23 mm, *y* = 0 mm, *z* = 28 mm]), D (**r** = [*x* =  − 23 mm, *y* =  − 5.5 mm, *z* = 27.5 mm]), E (**r** = [*x* =  − 23 mm, *y* = 5 mm, *z* = 28 mm]), and F (**r** = [*x* =  − 23 mm, *y* = 0 mm, *z* = 19 mm]), respectively. In (**c**)–(**h**), the white dotted rectangles denote the area where the waves scattered at A–F were concentrated, respectively. (**i**) Schematic illustrating the scattered waves for responses A and B. (**j**) Schematic illustrating the scattered waves for responses C, D, and E. (**k**) Schematic illustrating the scattered waves for response F.

After validating the high-resolution 3D imaging capability of PLUS, we applied the proposed methodology for 3D ultrasonic scattering analysis to the fatigue-crack specimen as schematically illustrated in Fig. 4a. Here, we selected several responses, A–F, from the collection of multiple responses (Fig. 3e), as shown in Fig. 4b, to examine the 3D scattered fields in detail. The color scales for the scattering intensities for A–F were fixed in Fig. 4c–h. Note that the fundamental validity of this analysis method for multiple responses has been reported in the Supplementary Material and the cautionary point is given later in Discussion.

For response A (Fig. 4c), we found that the scattered waves three-dimensionally diverged over the large area surrounded by the white dotted rectangle [*nx* = 20–71, *ny* = 1–57], as illustrated in Fig. 4i. This suggests that the large receiving aperture of PLUS with the ultra-multiple elements was required to receive all the scattered waves from A. A similar tendency was observed for response B (Fig. 4d), although the detail of the scattering-intensity pattern within the area surrounded by the white dotted rectangle was different from that in Fig. 4c. The complicated distributions in the *y*-direction in Fig. 4c, d may be due to the difference in the microscopic asperities of the crack faces in the vicinities of A and B. In contrast to A and B, the scattered waves from C, D, and E were concentrated in the narrower area [*nx* = 40–71, *ny* = 1–57] surrounded by the white dotted rectangles in Fig. 4e–g, as illustrated in Fig. 4j. This suggests that the waves scattered at C, D, and E had different directivities from those scattered at A and B. For response F, the scattered waves were further concentrated in the rightmost area [*nx* = 57–71, *ny* = 15–57] of the scan area, as shown in Fig. 4h. As illustrated in Fig. 4k, this shows that the waves scattered at F had stronger directivities than those scattered at A–E. It also suggests that the visualization of responses C–F requires the coverage of such local areas with the large receiving aperture of PLUS. Specifically, the accurate measurement of crack depth requires the visualization of the deepest part of the fatigue crack, i.e., the response at F, which was not visualized by PLUS with the small receiving aperture composed of 256 receiving points. This suggests that a small receiving aperture can overlook some parts of cracks, resulting in underestimation of the crack depth and length. The same problem can occur for a piezoelectric matrix array transducer with a limited number of elements. Although piezoelectric matrix array transducers are typically designed to avoid grating lobes^4,6^, the above findings will be useful for optimizing the inspection conditions and for the design of more sophisticated 2D array transducers for practical applications.

The above results also provide insight into characterization of the fatigue crack. Given that the incident wave was irradiated onto the crack from the left-hand side, most responses below the fatigue-crack tip in Fig. 3b, d were created by the forward-scattering waves. This cannot occur in a defect such as a slit with an air gap, except at the top of the slit. This suggests that the parts below the fatigue-crack tip had many contact points that could cause the forward scattering in the positive *x*-direction. On the other hand, we also found that the adjacent scattering sources within the fatigue crack exhibited different scattering behaviors. As shown in Fig. 4b, responses A, B, and F appeared in the vicinity of the deepest part of the crack. The spatial distances between these responses are similar. The scattering intensities (Fig. 4c, d) for responses A and B were similar, whereas response F exhibited significantly different 3D scattering behaviors as shown in Fig. 4h. This suggests that the crack characteristics can markedly change regardless of the spatial position within a crack, which could be one of the causes of the complexity of 3D ultrasonic scattering at cracks. Thus, the analyses of the 3D scattering intensities from respective responses could provide information on crack characteristics.

## Discussion

In the “Results” section, we found that the 3D scattering behaviors can drastically change depending on the responses within a single fatigue crack. On the other hand, the 3D scattering intensities were examined only for the oblique incidence of the transverse wave. As reported in past studies of 2D scatterings^18,20–22,50^, ultrasonic scatterings at cracks can involve mode conversion, such as from transverse waves to longitudinal waves. We can investigate mode-converted scattered waves at cracks in the same manner simply by selecting *V*_L_ instead of *V*_T_ as *V*_*j*_ in Eqs. (2)–(4). Furthermore, the incident angle can influence 3D ultrasonic scattering. We can investigate the incident-angle dependence of 3D ultrasonic scatterings by preparing the wedges with different angles. The advantage of the large receiving aperture of PLUS will be useful for measuring 3D scatterings for multiple modes and various incident angles. The systematic exploration of 3D ultrasonic scatterings at cracks can be used to optimize inspection conditions and may lead to the development of new ultrasonic measurement systems and the characterization of cracks.

The high-resolution 3D images (see Fig. 3) and 3D scattering analysis (see Fig. 4) clarified that the parts below the fatigue-crack tip had many contact points. This is surprising since the past studies based on 2D nonlinear ultrasonic imaging showed the possibility of only a few contact points below the crack tip^7,15,17,37,47,48^. On the other hand, the findings can also be useful for fracture mechanics. Thus far, three fatigue-crack closure mechanisms have been mainly proposed in fracture mechanics; plasticity-, roughness-, and oxide-induced crack closure (PICC, RICC, and OICC, respectively)^46^. PICC is caused by residual plastic strains in the wake of propagating fatigue cracks. OICC is caused by the oxide debris between crack faces. RICC is caused by the contact of rough crack faces. Given the complexity of the fatigue-crack closure induced by the mixture of the above mechanisms, fatigue cracks can be closed not only at the tip but also in the middle part. However, such evidence has yet to be reported because of the lack of measurement tools. The proposed technique can be a novel tool to elucidate fatigue-crack closure mechanisms in fracture mechanics.

On the other hand, each scattering-intensity distribution in Fig. 4 did not show the response only from a point scatterer, which can generate a spherical scattered wave. That is, the distributions showed the superposition of the scatterers in the vicinity of the center of each response. This led to the complicated scattering-intensity distributions shown in Fig. 4c–h. Also, note that the artifact due to the overlap of the waves scattered at other parts can influence the scattering-intensity distribution when there are a lot of scatterers, such as the fatigue cracks. Although such an issue can be reduced to a certain extent by selecting a short time window, as shown in Fig. S4 (Supplementary material), the perfect avoidance would be impossible. To suppress this problem, the utilization of focusing would be useful, as demonstrated for 2D scattering analysis^18^. For instance, the use of a point-focusing incidence instead of the plane-wave incidence would be effective since observable scatterers can be limited in the vicinity of the transmission focal point. Although this is beyond the scope of this study, it will be an important topic in future works.

Most importantly, the described methodology is very general because of the advantage of PLUS based on the LDV scan. PLUS utilizes the mechanical scan of an LDV that can receive broadband frequencies from 0 to 20 MHz, in contrast to a piezoelectric array transducer with a certain center frequency. Hence, an arbitrary frequency can be used by changing the piezoelectric transmitter, which is commercially available and has a relatively low cost. Furthermore, the area and pitch of the LDV can be flexibly changed depending on the object to be measured. This advantage enables the application of the proposed method to not only metals but also other materials such as plastics, concrete, rocks, and sandstones. Thus, the range of applicable fields is expected to be very diverse. For the detailed discussion on advantages of PLUS over a method using the mechanical scan of a piezoelectric array transducer, see the Supplementary Material. Furthermore, the fact that big data can be directly obtained by 3D elastic-wave scattering analysis should also open up new avenues in the use of machine learning^51,52^ to characterize interfaces and develop new methods.

Although we formulated Eqs. (1)–(4) for isotropic materials, the application of the proposed technique to composites, such as carbon fiber reinforced plastics (CFRP), is one of the challenging subjects. CFRP generally has strong elastic anisotropy, resulting in the direction dependence of the ultrasonic velocity and the beam skewing effect^53^. To apply the proposed technique to such anisotropic materials, Eqs. (1)–(4) should be modified by considering the elastic anisotropy^54,55^.

On the other hand, PLUS with the ultra-multiple receiving points has strong potential as a new tool for laboratory experiments and on-site applications. However, in this study, it took more than 6 h for PLUS to acquire the data while moving the receiving points over the scan area (i.e., 71 × 57 receiving points). This is a severe drawback for the on-site application of PLUS. Each acquisition was made after 64-times averaging in the oscilloscope to improve the SNR of the received waves. Instead of using an oscilloscope, a high-speed digitizer installed in a PC can shorten the acquisition time to approximately 1/10. On the other hand, the output power of the He–Ne LDV operated at a wavelength of 632.8 nm, which was employed in this study, was limited to 1 mW to stay in a safe laser class 2. In contrast, an infrared LDV operated at a wavelength of 1550 nm allows a higher output power of 10 mW in laser class 1 since the laser at the wavelength is absorbed in water very quickly and does not reach the retina of human eyes^56^. Replacing the He–Ne LDV employed in this study with an infrared LDV can markedly enhance the SNR, which can reduce the number of averaging. The acquisition time also depends on imaging volume, scan area, and scan pitch (i.e., the number of receiving points). An effective imaging volume is limited by the region that can be irradiated by incident ultrasonic waves. For instance, the fatigue crack close to the bottom and side surfaces was not visualized in Fig. 3c, e since we set the transmitter at the center of the specimen in the *y*-direction (See Fig. 3a) and intentionally irradiated the ultrasonic wave on the upper part of the fatigue crack to avoid missing the crack tip. We may be able to visualize a larger volume by using a diverging wave, whereas it can lower the SNR in 3D images. The balance between the effective imaging volume and SNR needs to be considered appropriately. Once the imaging volume has been determined, one needs to select a scan area and pitch. Figure 4 suggests that the scan area larger than the imaging region is desired to receive scattered waves sufficiently. For such a large scan area, the number of receiving points should be optimized by selecting an appropriate scan pitch. Furthermore, PLUS can utilize the concept of sparse 2D array^57–59^, since PLUS can realize arbitrary scan patterns without any additional cost. This can significantly reduce the number of receiving points. On the other hand, the measurement of using LDV often requires careful alignments to obtain a sufficient laser intensity returned from specimens. The retroreflective tape used in this study was effective for intensifying the laser reflection at the specimens. However, the use of retroreflective tape may not be allowable for practical application. In such a case, an infrared LDV with a higher power than the He–Ne LDV may solve the alignment issue without using such a tape, which could make PLUS more practical for on-site application.

## Conclusions

In this paper, we have proposed a method for analyzing 3D elastic-wave scattering at interfaces based on the high-resolution 3D imaging method using PLUS. The key idea is to take advantage of the high-resolution 3D imaging capability of PLUS and all the waveforms measured using the large-aperture 2D matrix array composed of ultra-multiple LDV receiving points. We have shown how the 3D elastic-wave scattering in the vicinity of each response can be extracted from the high-resolution 3D images obtained by PLUS. After confirming the fundamental performance of the proposed methodology in an FBH specimen, we experimentally applied it to a fatigue-crack specimen. The results showed the complicated 3D elastic-wave scatterings in the vicinity of each response within the single crack, which are unmeasurable by other methods. This is significant because access to 3D elastic-wave scattering would be useful for designing ultrasonic measurement systems, optimizing inspection conditions, and characterizing cracks. More importantly, the described methodology is very general and can be applied to not only metals but also composites, plastics, concrete, rocks, and sandstones, leading to progress in many research and industrial fields that utilize 3D elastic-wave scattering at interfaces. The combination of PLUS with nonlinear ultrasonics and guided waves will also be an interesting topic in future works.

## Supplementary Information

Supplementary Information.

## Data Availability

The datasets generated during the current study are available from the corresponding author on reasonable request.
