# Supplementary material for: Exploring 3D elastic-wave scattering at interfaces using high-resolution phased-array system
Source: Sci Rep. 2022 May 25;12:8291. doi: 10.1038/s41598-022-12104-9 (PMC9132965; doi:10.1038/s41598-022-12104-9)
Supplement: Supplementary file 1 — Supplementary Information. [file 41598_2022_12104_MOESM1_ESM.pdf]

SUPPLEMENTARY MATERIAL - Exploring 3D elastic-wave scattering at interfaces using high-resolution phased-array system

Yoshikazu Ohara[1]*, Marcel C. Remillieux[2], Timothy James Ulrich[2], Serina Ozawa[1], Kosuke Tsunoda[1], Toshihiro Tsuji[1], and Tsuyoshi Mihara[1]

[1]*Department of Materials Processing, Tohoku University, Sendai, Miyagi 980-8579, Japan*

[2]*Los Alamos National Laboratory, Los Alamos, New Mexico 87545, USA*

*E-mail: ohara@material.tohoku.ac.jp

Approximation in the formulation of 3D imaging algorithms

We used a simple approximation to formulate Eqs. (2) and (3). We believe that a fixed incidence point is reasonable given that the ultrasonic wave in the wedge would be a plane wave because of the small propagation distance. On the other hand, we can select either the approximation based on a plane wave or a spherical wave depending on experimental conditions. When the propagation distance is not so long (e.g., less than a Fresnel zone), the approximation of a plane-wave propagation would be suitable. Otherwise, the approximation of a spherical wave would be appropriate. In this experiment, either would be acceptable, considering the propagation distance to the FBH and fatigue crack. To demonstrate this, we made a script under the assumption of plane-wave incidence and compared those imaging algorithms in the FBH and fatigue-crack specimens. Figures S1 and S2 show the imaging results of the FBH and fatigue crack, respectively, obtained by the PLUS based on the different approximations. As a result, as expected, the images were almost the same, not depending on the approximations. This shows that either approximation would be acceptable in this experiment. Note that it is indispensable to consider which approximation is more suitable for different experimental conditions.

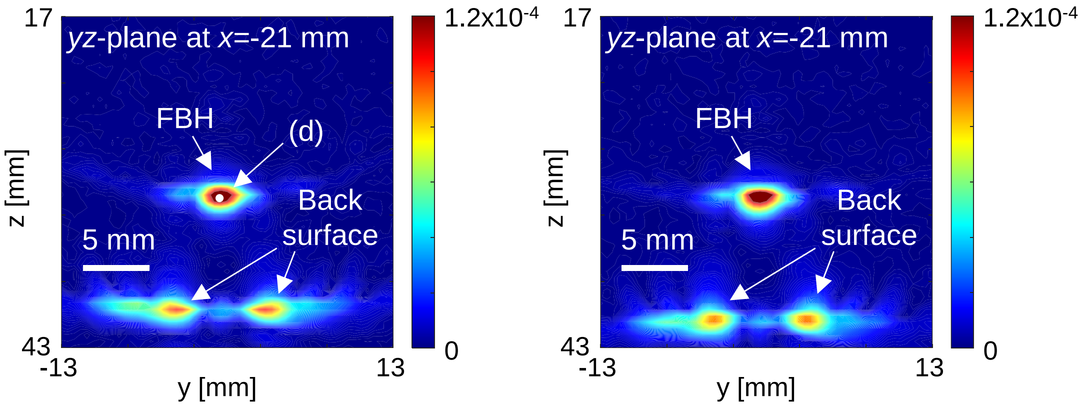

(a) Spherical-wave approximation (=Fig. 2(c))   (b) Plane-wave approximation

Figure S1. Imaging results of the flat bottom hole (FBH) by the PLUS using the imaging algorithms based on spherical-wave and plane-wave approximations.

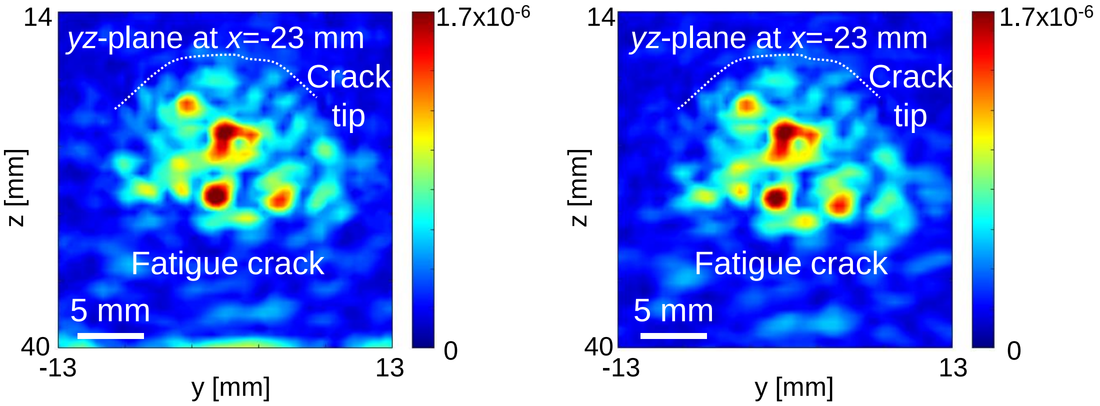

(a) Spherical-wave approximation (=Fig. 3(e))   (b) Plane-wave approximation

Figure S2. Imaging results of the fatigue crack by the PLUS using the imaging algorithms based on spherical-wave and plane-wave approximations.

Separation capability of multiple scattering sources in scattering-intensity distribution

We proposed a method of exploring 3D ultrasonic scattering based on our previously developed high-resolution 3D phased array system, the piezoelectric and laser ultrasonic system (PLUS).[1] We defined the scattering intensity for the response at a position vector **r** by the following equation:

$$I_{\mathrm{SC}}(\mathbf{r}, nx, ny) = \left(\frac{1}{\Delta\tau} \int_{t_{nx,ny}(\mathbf{r})}^{t_{nx,ny}(\mathbf{r})+\Delta\tau} u_{nx,ny}{}^{2}(t)\, dt \right)^{1/2} \tag{1}$$

where nx and ny are the indices of the receiving point in the x- and y-directions, respectively, $t_{nx,ny}(\mathbf{r})$ is the propagation time from the transmitter through $\mathbf{r}$ to the receiving point $\mathbf{r}_{nx,ny}$, $u_{nx,ny}(t)$ is the wave received at $\mathbf{r}_{nx,ny}$, and $\Delta\tau$ is the temporal window for the root mean square (RMS) calculation. Note that the scattering intensity in Eq.(1) comes from not only the position $\mathbf{r}$ but also other positions that have the same propagation distance between $\mathbf{r}$ and $\mathbf{r}_{nx,ny}$. It is not easy to perfectly cancel the artifact due to the scattered waves from positions different from r. On the other hand, we confirmed that the artifact was not significant because of the following reasons.

First, we considered two adjacent responses from which the propagation times should be similar. In Fig. 4(b), the fatigue-crack responses at B and F are very close to each other. However, the scattering-intensity distributions were rather different, as shown in Figs. 4(d) and 4(h), although the propagation times should also be close. This suggests that the influence of adjacent responses (i.e., B and F) on problematic artifacts is not significant.

We also examined the validity of Eq. (7) by following additional analyses. A key parameter in Eq. (7) is $\Delta\tau$. $\Delta\tau$ is the temporal window for the RMS calculation, which is used to produce scattering-intensity distribution. In this study, we set $\Delta\tau$ to 3T given the actual pulse length of 3 cycles, where T is the time period of the ultrasonic wave. If a sample has a single scatterer, such as the FBH, artifacts should not appear in $I_{\mathrm{SC}}(\mathbf{r}, nx, ny)$ regardless of $\Delta\tau$. On the other hand, if a sample has multiple scatterers, such as the fatigue crack, the increase in $\Delta\tau$ can cause artifacts in $I_{\mathrm{SC}}(\mathbf{r}, nx, ny)$ because the probability that the waves from scatterers at positions different from **r** are overlapped by lengthening the time between $\Delta t_{nx,ny}(\mathbf{r})$ and $\Delta t_{nx,ny}(\mathbf{r}) + \Delta\tau$ becomes high. In contrast, if $\Delta\tau$ is decreased, such overlap should be reduced.

To confirm these hypotheses, we varied $\Delta\tau$ to examine $I_{\mathrm{SC}}(\mathbf{r}, nx, ny)$ for the response [see Fig. 2] at the top surface of FBH. As shown in Fig. S3, $I_{\mathrm{SC}}(\mathbf{r}, nx, ny)$ did not change regardless of $\Delta\tau$. This is reasonable since the specimen has a single scatterer (i.e., the FBH). Note that artifacts did not appear for all the $\Delta\tau$, although the SNR became slightly low with the increase in $\Delta\tau$. This shows that the overlap of scattered waves from positions different

from **r** did not occur in the time between $\Delta t_{nx,ny}(\mathbf{r})$ and $\Delta t_{nx,ny}(\mathbf{r}) + \Delta\tau$.

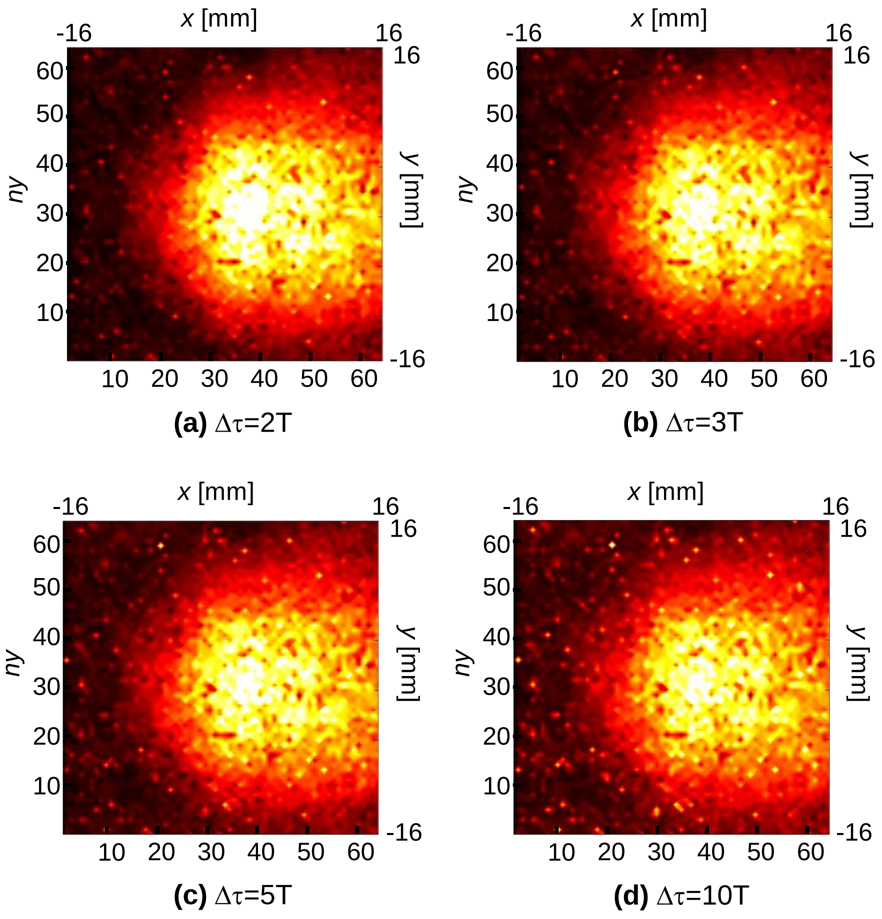

Figure S3. $\Delta\tau$ dependence of scattering-intensity distribution $I_{SC}(\mathbf{r}, nx, ny)$ for the FBH response at the point indicated as a white circle in Fig. 2(c). (a) $\Delta\tau$=2T, (b) $\Delta\tau$=3T, (c) $\Delta\tau$=5T, and (d) $\Delta\tau$=10T. (b) corresponds to Fig. 2(d).

Likewise, we varied Δτ to examine $I_{SC}(\mathbf{r}, nx, ny)$ for the fatigue-crack response at A shown in Fig. 4(b). In contrast to Figure S3, $I_{SC}(\mathbf{r}, nx, ny)$ markedly changed depending on Δτ, as shown in Figure S4. Specifically, as Δτ was increased from 3T, the bright region increased. When Δτ=10T, most of the area surrounded by the white dotted rectangle became bright in Fig. S4(d), which was significantly different from Fig. S4(b) (i.e., Fig. 4(c)). This is because many scattered waves from positions different from **r** (i.e., A) were overlapped in the time between $\Delta t_{nx,ny}(\mathbf{r})$ and $\Delta t_{nx,ny}(\mathbf{r}) + \Delta\tau$. In this case, the artifacts

would be a problem in investigating $I_{\mathrm{SC}}(\mathbf{r}, nx, ny)$. On the other hand, $\Delta\tau$ was decreased from 3T to 2T, the change in $I_{\mathrm{SC}}(\mathbf{r}, nx, ny)$ was small. This suggests that the overlap of the waves scattered at positions different from A was small in the time between $\Delta t_{nx,ny}(\mathbf{r})$ and $\Delta t_{nx,ny}(\mathbf{r}) + \Delta\tau$. Thus, we believe that the influence of the overlap on $I_{\mathrm{SC}}(\mathbf{r}, nx, ny)$ was not significant, although it was not easy to eliminate the artifacts perfectly.

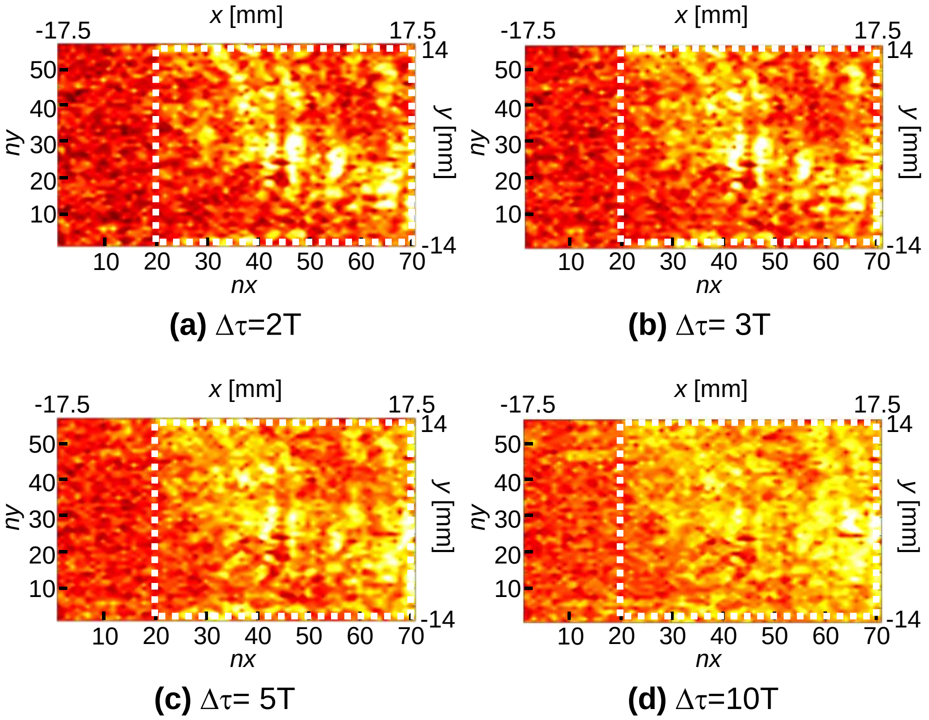

Figure S4. $\Delta\tau$ dependence of scattering-intensity distribution $I_{\mathrm{SC}}(\mathbf{r}, nx, ny)$ for the fatigue-crack response at A shown in Fig. 4(b). (a) $\Delta\tau$=2T, (b) $\Delta\tau$=3T, (c) $\Delta\tau$=5T, and (d) $\Delta\tau$=10T. (b) corresponds to Fig. 4(c).

Advantages of PLUS over a method using the mechanical scan of a piezoelectric array transducer

Although the proposed methodology is based on the LDV scan, a mechanical scan of a piezoelectric array transducer is an option to realize high-resolution 3D imaging and explore 3D ultrasonic scattering behaviors. Thus far, we have used the mechanical scan of a piezoelectric array receiver to realize a large-aperture array transducer, where we combined the mechanical scan of a piezoelectric 1D array receiver (32 elements) with a

monolithic piezoelectric transmitter.[2,3] In terms of the SNR, the mechanical scan of the LDV may be lower than that of a piezoelectric array transducer because of the small laser irradiation spot (i.e., tens of micron). In addition, PLUS requires the repetition of acquisition for all the receiving points, whereas a piezoelectric array transducer can acquire multiple received signals simultaneously. Nevertheless, PLUS still has advantages over a method using the mechanical scan of piezoelectric array transducer because of the following reasons. When using a piezoelectric array transducer, the element pitch is fixed. In contrast, PLUS can flexibly change the scan pitch. Although this study simulated typical 2D matrix arrays with a fixed pitch in the *x*- and *y*-directions, PLUS can also simulate sparse 2D array[4–6] to reduce the inspection time, which is unachievable for the mechanical scan of a piezoelectric 2D matrix array. On the other hand, the LDV used in this study has a broad reception bandwidth (0-20 MHz), and therefore, the frequency can be flexibly selected by changing the transmitter. For attenuative materials, a low frequency, such as less than 1 MHz, is suitable for 3D imaging. However, there is no commercially available low-frequency array transducer[7] because of the difficulty of avoiding the crosstalk between piezoelectric elements and dampening the vibration of each element. For more precise 3D imaging of small specimens, the use of a higher frequency, such as 15-20 MHz, would be desired. Such a high-frequency array transducer requires a very small element pitch, e.g., of less than a half wavelength, to avoid the generation of grating lobes.[8,9] For example, a half wavelength of the 15-MHz transverse wave in the aluminum alloy is approximately 0.1 mm, which may encounter the difficulty of fabricating such a 2D matrix array transducer, except for a capacitive micromachined ultrasonic transducer (CMUT).[10] In contrast, PLUS can realize such a small scan pitch since the laser irradiation spot is in the order of tens of microns. Although the mechanical scan of a piezoelectric array receiver can be used for a specific condition and shorten the acquisition time, the proposed methodology is very general and has the advantages described above over a method of using a piezoelectric array transducer.

Demonstration of the 3D imaging capability of PLUS for multiple scatterers by simulation

The theoretical demonstration of the 3D imaging capability of the proposed technique for

multiple scatterers is critically important. To this end, 3D numerical simulation, e.g., by finite element method (FEM), would be useful. However, such a simulation is not easy because of computation cost. Hence, we simulated the received waves at each acquisition point based on the propagation time $t_{nx,ny}(\mathbf{r}_{\mathbf{S}\boldsymbol{n}})$ given by Eq. (3) under the following assumption. We first assume that the incident wave is given by

$$\mathrm{s}(t) = \begin{cases} \left(0.5 - 0.5\cos\left(2\pi\frac{t}{\Delta\tau}\right)\right)\sin(2\pi f t) & 0 \le t \le \Delta\tau \\ 0 & t < 0, t > \Delta\tau \end{cases}, \qquad \text{(S1)}$$

and a scatterer generates a spherical scattered wave. For *n*th scatterer at $\mathbf{r}_{\mathbf{S}\boldsymbol{n}}$, a received waves $u_{nx,ny}(t)$ at an acquisition point $\mathbf{r}_{nx,ny}$ is expressed by

$$u_{nx,ny}(t) = s(t - t_{nx,ny}(\mathbf{r}_{\mathbf{S}\boldsymbol{n}}))\ , \qquad \text{(S2)}$$

where nx and ny are the indices of the receiving point in the x- and y-directions, respectively. When the number of scatterers is N, the received waves at $\mathbf{r}_{nx,ny}$ are expressed by

$$u_{nx,ny}(t) = \sum_{n=1}^{N} s(t - t_{nx,ny}(\mathbf{r}_{Sn}))\ . \qquad \text{(S3)}$$

Based on Eqs. (S1)-(S3) and (3), we simulated the received waves at each acquisition point.

Figure S5(a) shows a model to simulate the experimental conditions shown in Fig. 3(a). Incident and scattered waves propagate in an isotropic sample at a speed of V_T (3080 m/s) as transverse waves. For simplicity, no attenuation is considered. The incident wave was simulated using a Hanning-windowed burst wave (5 MHz, 3 cycles), as expressed by Eq. (S1). The number of receiving points was 4047 (i.e., 71×57). The pitch between the adjacent receiving points was fixed to 0.5 mm in the *x*- and *y*-directions. The imaging volume was 26×26×26 mm^3. The pitch of the imaging grid was set to 0.5 mm in the *x*-, *y*-, and *z*-directions. Here we varied the number of scatterers (1, 5, and 50), as shown in Figs. S5(b), S5(c), and S5(d), respectively. The single scatterer was set at a depth of 30 mm [see Table S1]. The 5 and 50 scatterers were randomly set within an imaging volume, as listed in Tables S2 and S3, respectively. On the above conditions, we made three datasets of scattered waves for 1, 5, and 50 scatterers, respectively, based on Eqs. (S1), (S2), and (S3).

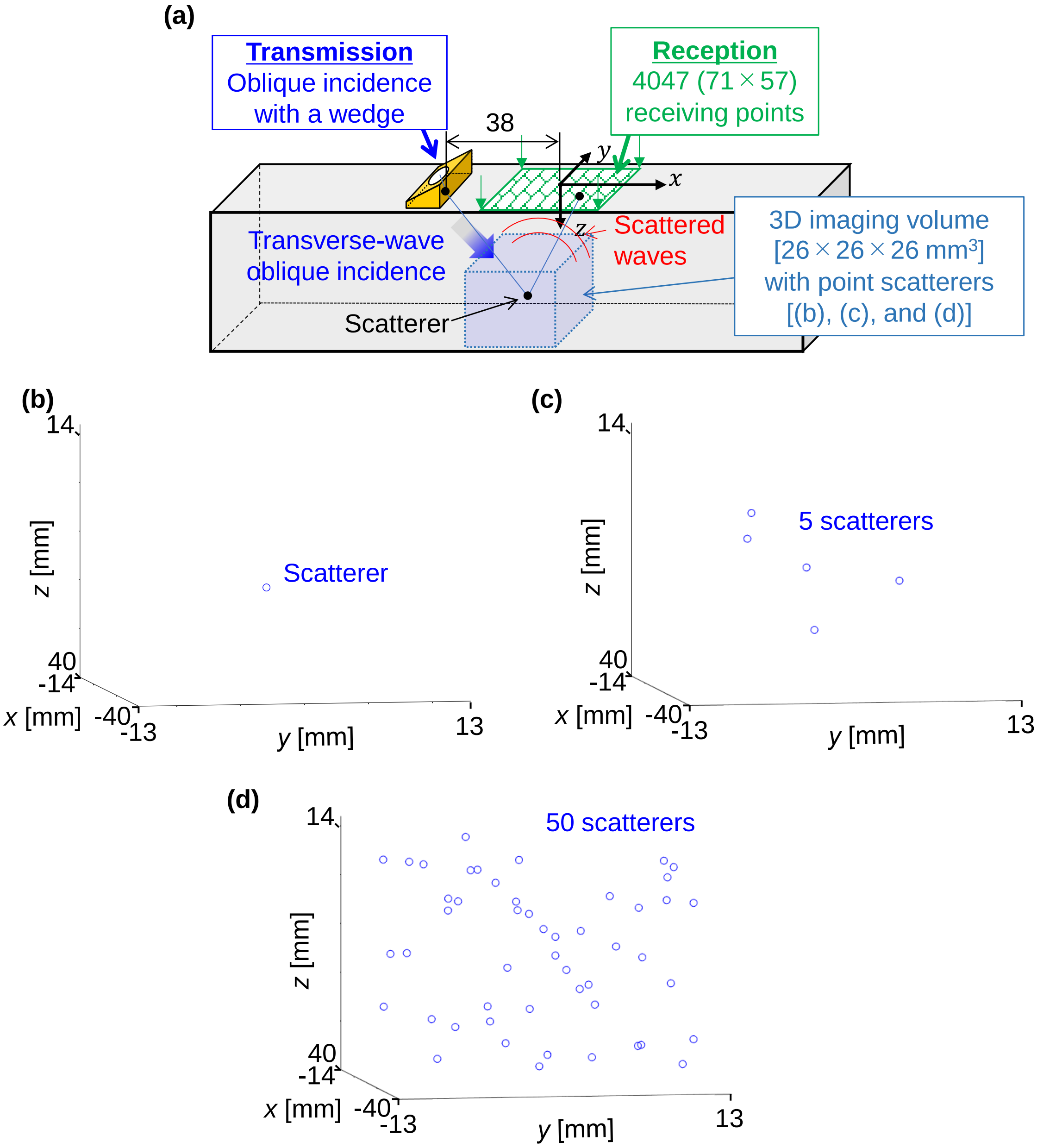

Figure S5. Simulation model. (a) A model with a condition to simulate the experiment for the fatigue crack [Fig. 3]. Distributions of (b) 1, (c) 5, and (d) 50 scatterers in the imaging volume.

No	*x* (mm)	*y* (mm)	*z* (mm)
1	−23.0	0	30.0

Table S1. Coordinate of the scatterer shown in Fig. S5(b).

No	*x* (mm)	*y* (mm)	*z* (mm)
1	−22.6	6.5	29.6
2	−29.8	−2.1	27.2
3	−24.6	−5.5	22.2
4	−30.8	−6.9	24.1
5	−26.1	−0.8	34.1

Table S2. Coordinates of the 5 scatterers shown in Fig. S5(c).

No	*x* (mm)	*y* (mm)	*z* (mm)	No	*x* (mm)	*y* (mm)	*z* (mm)
1	−35.8	6.6	35.3	26	−34.3	−1.9	21.9
2	−20.4	1.7	25.1	27	−34.1	10.2	37.6
3	−33.1	−0.8	37.7	28	−30.0	−5.6	17.8
4	−36.9	8.4	20.4	29	−27.4	10.7	18.1
5	−20.6	−3.5	18.9	30	−24.3	11.0	30.4
6	−22.4	−1.4	28.8	31	−18.9	−4.9	35.2
7	−25.1	−1.0	17.4	32	−33.7	11.1	21.1
8	−28.8	0.6	37.0	33	−19.3	9.6	28.3
9	−21.8	2.4	27.7	34	−23.4	−7.1	37.9
10	−20.9	7.3	27.0	35	−24.1	6.3	21.4
11	−34.1	−11.4	25.8	36	−28.7	1.8	22.2
12	−26.4	−2.3	36.1	37	−16.9	−5.1	22.3
13	−34.3	−4.5	18.7	38	−26.6	−11.9	17.0
14	−37.8	2.2	28.8	39	−20.7	0.6	33.2
15	−17.4	−4.4	22.5	40	−34.5	8.9	18.3
16	−34.6	−5.0	32.8	41	−18.5	−6.7	34.4
17	−16.5	−3.7	16.0	42	−20.0	−2.6	33.0
18	−27.0	4.3	37.6	43	−29.5	2.9	30.2
19	−17.3	3.2	26.3	44	−19.6	−10.6	32.9
20	−20.8	−8.8	18.0	45	−19.2	−7.4	18.4
21	−23.2	5.2	32.6	46	−33.0	−8.0	21.6
22	−31.1	1.6	28.1	47	−24.2	1.1	21.8
23	−30.3	11.7	35.5	48	−16.4	−9.5	27.9
24	−30.6	7.4	21.9	49	−29.2	3.1	24.3
25	−29.0	7.6	36.2	50	−23.3	10.6	18.0

Table S3. Coordinates of the 50 scatterers shown in Fig. S5(d).

We first carried out the simulation for the single scatterer [Fig. S5(b)]. Following the above equations, we made a dataset of received waves at 4047 (i.e., 71×57) receiving points. As an example, Fig. S6(a) shows the wave received at nx=28 and ny=34. The received wave had a single scattered wave since the single scatterer existed. We applied the

3D imaging algorithm used in this study for the experiments to the dataset for the single scatterer. Figure S6(b) shows the 3D imaging results. The point scatterer was visualized at the correct position.

In the model with the 5 scatterers [Fig. S5(c)]. As shown in Fig. S7(a), the received wave at *nx*=28 and *ny*=34 had five scattered waves, which were not overlapped in the time domain. Using the dataset of the received waves, we performed the 3D imaging processing. As a result, the 5 scatterers were visualized at the correct positions, as shown in Fig. S7(b). Note that no artifact appeared in the imaging result.

In the model with the 50 scatterers [Fig. S5(d)], the received wave at *nx*=28 and *ny*=34 was composed of 50 scattered waves, which were complexly overlapped, as shown in Fig. S8(a). Using the dataset of the received waves, we performed the 3D imaging process. Although the received waves were complicated because of the overlap of the waves scattered from the 50 scatterers, the scatterers were successfully visualized at the correct positions, as shown in Fig. S8(b). Note that the positions of the scatterers visualized in Fig. S8(b) are in good agreement with those in Fig. S5(d). Importantly, no artifact was observed in the imaging result. This shows that the delay-and-sum processing using ultra-multiple (i.e., 4047) received waves has a powerful 3D imaging capability for the 50 scatterers. Note that the above model does not consider the multiple scatterings among the scatterers and the mode conversion[11] at scatterings, which can cause artifacts. However, given the simple geometries of the FBH and fatigue crack used in this study, it is reasonable that such multiple scattering and mode conversion did not occur in the experiments. Thus, the above simulation demonstrated the 3D imaging capability of PLUS and the validity of the 3D imaging results [Figs. 2 and 3].

On the other hand, the examination of the scattering intensity at one of them can be influenced by other scatterers since the partial overlaps of the scattered waves from the 50 scatterers are inevitable given the complicated received waveforms [Fig. S8(a)]. This may cause the artifact in a scattering-intensity distribution. To suppress such an artifact, the utilization of focusing would be useful, as demonstrated for precise 2D scattering analysis.[12] The use of a monolithic point focusing transducer or a piezoelectric 2D matrix array for transmission focusing would enable us to investigate more precise 3D scattering intensity for the scatterers in the vicinity of a transmission focal point. This is one of

exciting and important future works, although this is beyond the scope of this study.

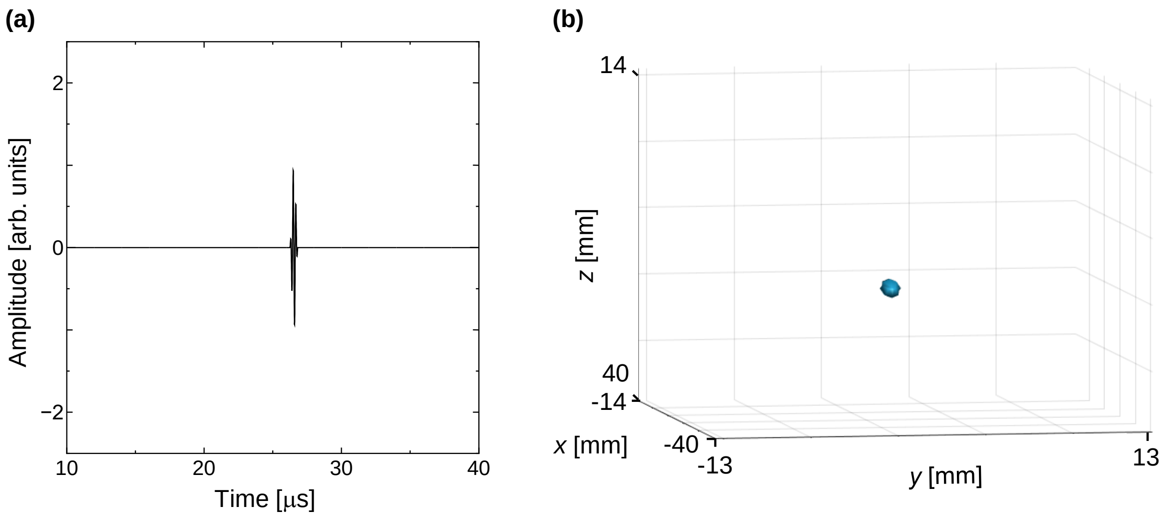

Figure S6. Simulation results of received wave and 3D image for a scatterer at the position shown in Table S1. (a) Received wave at *nx*=28 and *ny*=34. (b) 3D image obtained by PLUS.

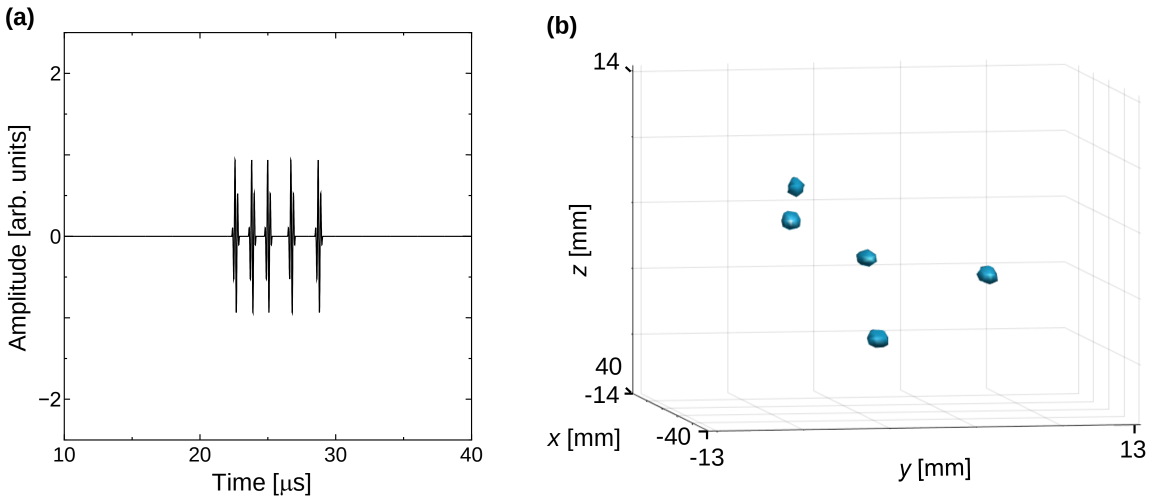

Figure S7. Simulation results of received wave and 3D image for 5 scatterers at the positions listed in Table S2. (a) Received wave at *nx*=28 and *ny*=34. (b) 3D image obtained by PLUS.

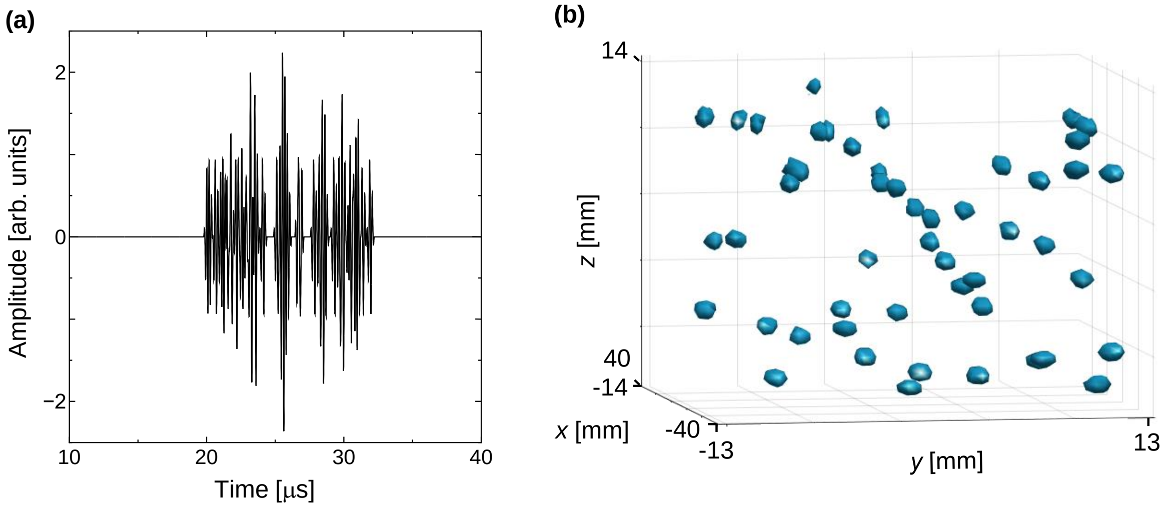

Figure S8. Simulation results of received waveform and 3D image for 50 scatterers at the positions listed in Table S3. (a) Received waveform at *nx*=28 and *ny*=34. (b) 3D image obtained by PLUS.

References

1. Ohara, Y. *et al.* Toward an ultra-high resolution phased-array system for 3D ultrasonic imaging of solids. *Appl. Phys. Lett.* **117**, 111902-1–5 (2020).
2. Ohara, Y., Endo, H., Mihara, T. & Yamanaka, K. Ultrasonic measurement of closed stress corrosion crack depth using subharmonic phased array. *Jpn. J. Appl. Phys.* **48**, 07GD01-1–6 (2009).
3. Ohara, Y., Potter, J., Nakajima, H., Tsuji, T. & Mihara, T. Multi-mode nonlinear ultrasonic phased array for imaging closed cracks. *Jpn. J. Appl. Phys.* **58**, SGGB06-1–7 (2019).
4. Davidsen, R. E., Jensen, J. A. & Smith, S. W. Two-dimensional random arrays for real time volumetric imaging. *Ultrasonic Imaging* vol. 16 143–163 (1994).
5. Austeng, A. & Holm, S. Sparse 2-D arrays for 3-D phased array imaging - Design methods. *IEEE Trans. Ultrason. Ferroelectr. Freq. Control* **49**, 1073–1086 (2002).
6. Roux, E. *et al.* Experimental 3-D Ultrasound Imaging with 2-D Sparse Arrays using Focused and Diverging Waves. *Sci. Rep.* **8**, 1–12 (2018).
7. Ohara, Y., Kikuchi, K., Tsuji, T. & Mihara, T. Development of low-frequency

phased array for imaging defects in concrete structures. *Sensors* **21**, 7012-1–15 (2021).

8. Schmerr, L. W. *Fundamentals of Ultrasonic Phased Arrays*. vol. 215 (Springer International Publishing, 2015).
9. Pompei, F. J. & Wooh, S.-C. Phased array element shapes for suppressing grating lobes. *J. Acoust. Soc. Am.* **111**, 2040 (2002).
10. Bhuyan, A. *et al.* Integrated circuits for volumetric ultrasound imaging with 2-D CMUT arrays. *IEEE Trans. Biomed. Circuits Syst.* **7**, 796–804 (2013).
11. Ohara, Y., Potter, J., Nakajima, H., Tsuji, T. & Mihara, T. Multi-mode nonlinear ultrasonic phased array for imaging closed cracks. *Jpn. J. Appl. Phys.* **58**, SGGB06-1–7 (2019).
12. Villaverde, E. L., Croxford, A. J. & Velichko, A. Optimal extraction of ultrasonic scattering features in coarse grained materials. *IEEE Trans. Ultrason. Ferroelectr. Freq. Control* **68**, 2238–2250 (2021).
